# Supplementary material for: Altered microRNA Transcriptome in Cultured Human Liver Cells upon Infection with Ebola Virus
Source: Int J Mol Sci. 2021 Apr 6;22(7):3792. doi: 10.3390/ijms22073792 (PMC8038836; doi:10.3390/ijms22073792)
Supplement: Supplementary file 1 [file ijms-22-03792-s001.zip › Supplementary_File/C_ GO_Analysis_Results/16-30nt_go_Reston-96h-Huh7_vs_Control-96h-Huh7_up.mature_mirna_targets/BP_result(Human).html]

| GO.ID | Term | Ontology | Count | Pop.Hits | List.Total | Pop.Total | Fold.Enrichment | Pvalue | FDR | Enrichment.Score | Gene.Ratio | GENES |
| --- | --- | --- | --- | --- | --- | --- | --- | --- | --- | --- | --- | --- |
| GO:0034612 | response to tumor necrosis factor | Biological process | 8 | 305 | 100 | 17653 | 4.63029508196721 | 0.000340428583929012 | 1 | 3.46797398172063 | 0.08 | CLIP3//TNFRSF11B//PSMD13//TNFRSF8//MAPK3//ZFP36L1//MAP2K7//CASP3// |
| GO:0006970 | response to osmotic stress | Biological process | 4 | 69 | 100 | 17653 | 10.2336231884058 | 0.000631996472147987 | 1 | 3.19928534597543 | 0.04 | CASP3//ZFP36L1//FMO1//MAP2K7// |
| GO:0030866 | cortical actin cytoskeleton organization | Biological process | 3 | 40 | 100 | 17653 | 13.23975 | 0.0014967424996294 | 1 | 2.82485290948275 | 0.03 | WIPF2//RTKN2//PLS1// |
| GO:0060627 | regulation of vesicle-mediated transport | Biological process | 9 | 490 | 100 | 17653 | 3.24238775510204 | 0.00185036537682824 | 1 | 2.73274250647669 | 0.09 | LRRTM1//SYT16//GRIK5//CLIP3//CFTR//CAMK1D//SYN1//SMAP1//MAPK3// |
| GO:0045619 | regulation of lymphocyte differentiation | Biological process | 5 | 156 | 100 | 17653 | 5.65801282051282 | 0.00193861865776184 | 1 | 2.71250761181742 | 0.05 | NFKBID//ZFP36L1//CYP26B1//NFATC2//NDFIP1// |
| GO:0008298 | intracellular mRNA localization | Biological process | 2 | 12 | 100 | 17653 | 29.4216666666667 | 0.00202065715033954 | 1 | 2.69450736800219 | 0.02 | PRPF18//ZFP36L1// |
| GO:0050864 | regulation of B cell activation | Biological process | 5 | 158 | 100 | 17653 | 5.58639240506329 | 0.00204957249023033 | 1 | 2.6883367167472 | 0.05 | CASP3//NFATC2//ZFP36L1//NDFIP1//IGLL5// |
| GO:0042487 | regulation of odontogenesis of dentin-containing tooth | Biological process | 2 | 14 | 100 | 17653 | 25.2185714285714 | 0.00276556264442785 | 1 | 2.55821649961709 | 0.02 | TNFRSF11B//BMP2// |
| GO:0030865 | cortical cytoskeleton organization | Biological process | 3 | 50 | 100 | 17653 | 10.5918 | 0.0028502649301172 | 1 | 2.54511477074919 | 0.03 | WIPF2//RTKN2//PLS1// |
| GO:0043065 | positive regulation of apoptotic process | Biological process | 10 | 627 | 100 | 17653 | 2.81547049441786 | 0.00291683175528037 | 1 | 2.53508862053908 | 0.1 | CASP3//GRIK5//NFKBID//FAP//SKIL//TFAP4//CLIP3//CAMK1D//BMP2//TNFRSF8// |
| GO:0071466 | cellular response to xenobiotic stimulus | Biological process | 5 | 174 | 100 | 17653 | 5.07270114942529 | 0.00311115195029392 | 1 | 2.50707877720289 | 0.05 | FMO1//CYP26B1//RORC//CDO1//TFAP4// |
| GO:0043068 | positive regulation of programmed cell death | Biological process | 10 | 633 | 100 | 17653 | 2.78878357030016 | 0.00312213884468025 | 1 | 2.50554778732816 | 0.1 | CASP3//CLIP3//CAMK1D//BMP2//TFAP4//TNFRSF8//GRIK5//NFKBID//FAP//SKIL// |
| GO:1904355 | positive regulation of telomere capping | Biological process | 2 | 17 | 100 | 17653 | 20.7682352941176 | 0.00408767444006144 | 1 | 2.38852370057273 | 0.02 | MAPK3//MAP2K7// |
| GO:0048534 | hematopoietic or lymphoid organ development | Biological process | 12 | 882 | 100 | 17653 | 2.40176870748299 | 0.00413500077557781 | 1 | 2.38352440465336 | 0.12 | NDFIP1//RTKN2//BMI1//CASP3//ZFP36L1//NFKBID//CYP26B1//NFATC2//SMAP1//MAPK3//RORC//PSMD13// |
| GO:0045600 | positive regulation of fat cell differentiation | Biological process | 3 | 60 | 100 | 17653 | 8.8265 | 0.00477740689091276 | 1 | 2.32080776835602 | 0.03 | CREBL2//BMP2//ZFP36L1// |
| GO:0006805 | xenobiotic metabolic process | Biological process | 4 | 120 | 100 | 17653 | 5.88433333333333 | 0.00482173228753874 | 1 | 2.31679690620914 | 0.04 | CDO1//FMO1//CYP26B1//RORC// |
| GO:0045598 | regulation of fat cell differentiation | Biological process | 4 | 121 | 100 | 17653 | 5.83570247933884 | 0.00496524963680788 | 1 | 2.30405891168769 | 0.04 | CREBL2//BMP2//ZFP36L1//RORC// |
| GO:0006833 | water transport | Biological process | 2 | 19 | 100 | 17653 | 18.5821052631579 | 0.00510192192838355 | 1 | 2.2922661914204 | 0.02 | CFTR//SLC14A1// |
| GO:0010942 | positive regulation of cell death | Biological process | 10 | 681 | 100 | 17653 | 2.59221732745962 | 0.00521398023760064 | 1 | 2.28283061925846 | 0.1 | CASP3//CLIP3//CAMK1D//BMP2//TFAP4//TNFRSF8//GRIK5//NFKBID//FAP//SKIL// |
| GO:0071356 | cellular response to tumor necrosis factor | Biological process | 6 | 287 | 100 | 17653 | 3.69052264808362 | 0.0058363324800843 | 1 | 2.23385997549123 | 0.06 | CLIP3//TNFRSF11B//PSMD13//TNFRSF8//MAPK3//ZFP36L1// |
| GO:0002520 | immune system development | Biological process | 12 | 928 | 100 | 17653 | 2.28271551724138 | 0.00616230808503778 | 1 | 2.21025659289231 | 0.12 | NDFIP1//RTKN2//BMI1//CASP3//ZFP36L1//NFKBID//CYP26B1//NFATC2//SMAP1//MAPK3//RORC//PSMD13// |
| GO:0018230 | peptidyl-L-cysteine S-palmitoylation | Biological process | 2 | 22 | 100 | 17653 | 16.0481818181818 | 0.00681640412018632 | 1 | 2.16644466971374 | 0.02 | CLIP3//ZDHHC11// |
| GO:0018231 | peptidyl-S-diacylglycerol-L-cysteine biosynthetic process from peptidyl-cysteine | Biological process | 2 | 22 | 100 | 17653 | 16.0481818181818 | 0.00681640412018632 | 1 | 2.16644466971374 | 0.02 | CLIP3//ZDHHC11// |
| GO:0045444 | fat cell differentiation | Biological process | 5 | 210 | 100 | 17653 | 4.20309523809524 | 0.00686384463545996 | 1 | 2.16343255539333 | 0.05 | RORC//CREBL2//BMP2//ZFP36L1//CLIP3// |
| GO:0048522 | positive regulation of cellular process | Biological process | 41 | 5142 | 100 | 17653 | 1.4075709840529 | 0.00722312065579551 | 1 | 2.14127513057671 | 0.41 | DUSP9//MAPK3//MAP2K7//BMP2//CLIP3//CASP3//ESM1//RTKN2//SHC4//CHN1//MSR1//CAMK1D//CREBL2//NFATC2//RORC//TFAP4//NFE2L3//NDFIP1//ARFIP1//CFTR//TNFRSF8//GRIK5//ZFP36L1//SMAP1//DMRT2//DLX3//TEAD3//BMI1//SKIL//IGLL5//WIPF2//LRRTM1//ACPP//DND1//NFKBID//FAP//PSMD13//GLS2//MIS18A//DPH6//CYP26B1// |
| GO:0030097 | hemopoiesis | Biological process | 11 | 842 | 100 | 17653 | 2.30621140142518 | 0.00806648873257873 | 1 | 2.09331546848859 | 0.11 | NDFIP1//CASP3//ZFP36L1//NFKBID//CYP26B1//NFATC2//SMAP1//RORC//PSMD13//RTKN2//BMI1// |
| GO:0071901 | negative regulation of protein serine/threonine kinase activity | Biological process | 4 | 142 | 100 | 17653 | 4.97267605633803 | 0.00867311318480164 | 1 | 2.06182498599593 | 0.04 | DUSP18//DUSP9//TFAP4//CASP3// |
| GO:2000300 | regulation of synaptic vesicle exocytosis | Biological process | 2 | 25 | 100 | 17653 | 14.1224 | 0.00875541031330867 | 1 | 2.05772349633893 | 0.02 | GRIK5//SYN1// |
| GO:0051384 | response to glucocorticoid | Biological process | 4 | 143 | 100 | 17653 | 4.9379020979021 | 0.00888467481988977 | 1 | 2.0513584627953 | 0.04 | ZFP36L1//TFAP4//CDO1//CASP3// |
| GO:0030217 | T cell differentiation | Biological process | 5 | 225 | 100 | 17653 | 3.92288888888889 | 0.00910109396389819 | 1 | 2.04090640175261 | 0.05 | ZFP36L1//NFKBID//CYP26B1//NFATC2//RORC// |
| GO:0017158 | regulation of calcium ion-dependent exocytosis | Biological process | 3 | 76 | 100 | 17653 | 6.96828947368421 | 0.00919630825494142 | 1 | 2.03638647985857 | 0.03 | GRIK5//SYN1//SYT16// |
| GO:0000188 | inactivation of MAPK activity | Biological process | 2 | 26 | 100 | 17653 | 13.5792307692308 | 0.00945026457389182 | 1 | 2.02455603261531 | 0.02 | DUSP18//DUSP9// |
| GO:0042044 | fluid transport | Biological process | 2 | 26 | 100 | 17653 | 13.5792307692308 | 0.00945026457389182 | 1 | 2.02455603261531 | 0.02 | SLC14A1//CFTR// |
| GO:0042481 | regulation of odontogenesis | Biological process | 2 | 26 | 100 | 17653 | 13.5792307692308 | 0.00945026457389182 | 1 | 2.02455603261531 | 0.02 | BMP2//TNFRSF11B// |
| GO:1904353 | regulation of telomere capping | Biological process | 2 | 26 | 100 | 17653 | 13.5792307692308 | 0.00945026457389182 | 1 | 2.02455603261531 | 0.02 | MAPK3//MAP2K7// |
| GO:0000187 | activation of MAPK activity | Biological process | 4 | 147 | 100 | 17653 | 4.80353741496599 | 0.0097646117866233 | 1 | 2.01034501835449 | 0.04 | MAP2K7//DUSP9//MAPK3//BMP2// |
| GO:0051125 | regulation of actin nucleation | Biological process | 2 | 28 | 100 | 17653 | 12.6092857142857 | 0.0109110164919168 | 1 | 1.96213478779239 | 0.02 | ARFIP1//WIPF2// |
| GO:1902803 | regulation of synaptic vesicle transport | Biological process | 2 | 28 | 100 | 17653 | 12.6092857142857 | 0.0109110164919168 | 1 | 1.96213478779239 | 0.02 | GRIK5//SYN1// |
| GO:0008652 | cellular amino acid biosynthetic process | Biological process | 3 | 81 | 100 | 17653 | 6.53814814814815 | 0.0109371320402127 | 1 | 1.96109654476673 | 0.03 | CDO1//GLS2//SEPHS2// |
| GO:0030098 | lymphocyte differentiation | Biological process | 6 | 330 | 100 | 17653 | 3.20963636363636 | 0.011196907234633 | 1 | 1.95090191986717 | 0.06 | ZFP36L1//NFKBID//CYP26B1//NFATC2//NDFIP1//RORC// |
| GO:1903955 | positive regulation of protein targeting to mitochondrion | Biological process | 3 | 83 | 100 | 17653 | 6.38060240963855 | 0.0116831321959798 | 1 | 1.93244070918686 | 0.03 | GLS2//MIS18A//DPH6// |
| GO:0042475 | odontogenesis of dentin-containing tooth | Biological process | 3 | 85 | 100 | 17653 | 6.23047058823529 | 0.0124578024003619 | 1 | 1.90455856197633 | 0.03 | BMP2//TNFRSF11B//DLX3// |
| GO:0012501 | programmed cell death | Biological process | 20 | 2088 | 100 | 17653 | 1.69090038314176 | 0.0124681052410728 | 1 | 1.90419954057676 | 0.2 | SHC4//XAF1//TMEM214//MAPK3//MAP2K7//ZFP36L1//CASP3//CAMK1D//GLS2//TNFRSF11B//BMP2//TNFRSF8//CLIP3//TFAP4//GRIK5//NFKBID//CYP26B1//ZMAT4//FAP//SKIL// |
| GO:0022408 | negative regulation of cell-cell adhesion | Biological process | 4 | 159 | 100 | 17653 | 4.44100628930818 | 0.0127384347928898 | 1 | 1.89488393170015 | 0.04 | NFKBID//NDFIP1//CASP3//BMP2// |
| GO:0033993 | response to lipid | Biological process | 11 | 900 | 100 | 17653 | 2.15758888888889 | 0.012866371555043 | 1 | 1.89054391119968 | 0.11 | MAPK3//CASP3//FMO1//TNFRSF11B//TNFRSF8//RORC//CDO1//CYP26B1//ZFP36L1//TFAP4//CFTR// |
| GO:0031960 | response to corticosteroid | Biological process | 4 | 160 | 100 | 17653 | 4.41325 | 0.0130095459416557 | 1 | 1.88573786089226 | 0.04 | CDO1//CASP3//ZFP36L1//TFAP4// |
| GO:0038093 | Fc receptor signaling pathway | Biological process | 5 | 246 | 100 | 17653 | 3.5880081300813 | 0.013015112340861 | 1 | 1.88555207889509 | 0.05 | NFATC2//MAPK3//MAP2K7//PSMD13//WIPF2// |
| GO:0006915 | apoptotic process | Biological process | 19 | 1958 | 100 | 17653 | 1.71300817160368 | 0.0131273945094412 | 1 | 1.88182146297819 | 0.19 | CASP3//CAMK1D//GLS2//TNFRSF11B//MAP2K7//BMP2//TNFRSF8//CLIP3//TFAP4//GRIK5//NFKBID//ZMAT4//FAP//SKIL//ZFP36L1//SHC4//XAF1//TMEM214//MAPK3// |
| GO:0045736 | negative regulation of cyclin-dependent protein serine/threonine kinase activity | Biological process | 2 | 31 | 100 | 17653 | 11.3890322580645 | 0.0132754977074321 | 1 | 1.87694918794597 | 0.02 | TFAP4//CASP3// |
| GO:0050869 | negative regulation of B cell activation | Biological process | 2 | 31 | 100 | 17653 | 11.3890322580645 | 0.0132754977074321 | 1 | 1.87694918794597 | 0.02 | CASP3//NDFIP1// |
| GO:0051650 | establishment of vesicle localization | Biological process | 5 | 249 | 100 | 17653 | 3.54477911646586 | 0.0136542345981266 | 1 | 1.86473263965656 | 0.05 | SYT16//GRIK5//DYNC1I1//F5//SYN1// |
| GO:0016079 | synaptic vesicle exocytosis | Biological process | 3 | 88 | 100 | 17653 | 6.01806818181818 | 0.0136739244310062 | 1 | 1.8641068245594 | 0.03 | GRIK5//SYT16//SYN1// |
| GO:1903706 | regulation of hemopoiesis | Biological process | 7 | 450 | 100 | 17653 | 2.74602222222222 | 0.0140259648614756 | 1 | 1.85306725345138 | 0.07 | NDFIP1//NFKBID//ZFP36L1//CYP26B1//NFATC2//SMAP1//PSMD13// |
| GO:0071470 | cellular response to osmotic stress | Biological process | 2 | 32 | 100 | 17653 | 11.033125 | 0.0141087677384128 | 1 | 1.85051091592514 | 0.02 | CASP3//ZFP36L1// |
| GO:0018345 | protein palmitoylation | Biological process | 2 | 33 | 100 | 17653 | 10.6987878787879 | 0.0149641336857374 | 1 | 1.82494842058084 | 0.02 | CLIP3//ZDHHC11// |
| GO:1904030 | negative regulation of cyclin-dependent protein kinase activity | Biological process | 2 | 33 | 100 | 17653 | 10.6987878787879 | 0.0149641336857374 | 1 | 1.82494842058084 | 0.02 | TFAP4//CASP3// |
| GO:0051249 | regulation of lymphocyte activation | Biological process | 7 | 457 | 100 | 17653 | 2.70396061269147 | 0.0151491054185759 | 1 | 1.81961301226049 | 0.07 | CASP3//NFATC2//NFKBID//NDFIP1//ZFP36L1//CYP26B1//IGLL5// |
| GO:1902105 | regulation of leukocyte differentiation | Biological process | 5 | 256 | 100 | 17653 | 3.4478515625 | 0.0152272497668444 | 1 | 1.81737852863637 | 0.05 | NDFIP1//NFKBID//ZFP36L1//CYP26B1//NFATC2// |
| GO:0007166 | cell surface receptor signaling pathway | Biological process | 26 | 3016 | 100 | 17653 | 1.52181034482759 | 0.0158208576337693 | 1 | 1.80076997750599 | 0.26 | CLEC4C//PSMD13//BMP2//SHC4//REPS2//SKIL//MAPK3//CLIP3//LRRTM1//RORC//CASP3//TNFRSF11B//TNFRSF8//GRIK5//NFATC2//MAP2K7//WIPF2//CHN1//IGLL5//CHRNA1//GLRB//ACPP//XAF1//MKS1//ESM1//VIPR2// |
| GO:0032212 | positive regulation of telomere maintenance via telomerase | Biological process | 2 | 34 | 100 | 17653 | 10.3841176470588 | 0.0158413237223749 | 1 | 1.80020853099758 | 0.02 | MAPK3//MAP2K7// |
| GO:1903214 | regulation of protein targeting to mitochondrion | Biological process | 3 | 93 | 100 | 17653 | 5.69451612903226 | 0.0158462679932506 | 1 | 1.80007300352708 | 0.03 | GLS2//MIS18A//DPH6// |
| GO:0042110 | T cell activation | Biological process | 7 | 463 | 100 | 17653 | 2.66892008639309 | 0.0161618480283467 | 1 | 1.79150898127025 | 0.07 | CYP26B1//ZFP36L1//NFKBID//NDFIP1//NFATC2//CASP3//RORC// |
| GO:0045165 | cell fate commitment | Biological process | 5 | 260 | 100 | 17653 | 3.39480769230769 | 0.0161785488979438 | 1 | 1.79106043413767 | 0.05 | CYP26B1//TEAD3//RORC//BMP2//CASP3// |
| GO:0051648 | vesicle localization | Biological process | 5 | 261 | 100 | 17653 | 3.38180076628352 | 0.0164224322740649 | 1 | 1.78456252048231 | 0.05 | SYT16//GRIK5//DYNC1I1//F5//SYN1// |
| GO:0033209 | tumor necrosis factor-mediated signaling pathway | Biological process | 4 | 173 | 100 | 17653 | 4.08161849710983 | 0.0168731525762521 | 1 | 1.77280376634054 | 0.04 | CLIP3//TNFRSF11B//PSMD13//TNFRSF8// |
| GO:0006469 | negative regulation of protein kinase activity | Biological process | 5 | 263 | 100 | 17653 | 3.35608365019011 | 0.0169175375094615 | 1 | 1.77166285192834 | 0.05 | DUSP18//DUSP9//TFAP4//CASP3//LRRTM1// |
| GO:0060341 | regulation of cellular localization | Biological process | 10 | 815 | 100 | 17653 | 2.16601226993865 | 0.0170592252571974 | 1 | 1.76804069615585 | 0.1 | GRIK5//CFTR//SYN1//CLIP3//PLS1//GLS2//MIS18A//DPH6//ZFP36L1//MAPK3// |
| GO:0051973 | positive regulation of telomerase activity | Biological process | 2 | 36 | 100 | 17653 | 9.80722222222222 | 0.0176601003206682 | 1 | 1.75300683368094 | 0.02 | MAPK3//MAP2K7// |
| GO:1904358 | positive regulation of telomere maintenance via telomere lengthening | Biological process | 2 | 36 | 100 | 17653 | 9.80722222222222 | 0.0176601003206682 | 1 | 1.75300683368094 | 0.02 | MAPK3//MAP2K7// |
| GO:0051591 | response to cAMP | Biological process | 3 | 97 | 100 | 17653 | 5.45969072164948 | 0.0177160702112916 | 1 | 1.75163260721342 | 0.03 | CFTR//ZFP36L1//CDO1// |
| GO:0051403 | stress-activated MAPK cascade | Biological process | 5 | 267 | 100 | 17653 | 3.30580524344569 | 0.0179373681298999 | 1 | 1.74624127871175 | 0.05 | DUSP9//MAP2K7//MAPK3//ZFP36L1//BMP2// |
| GO:0010831 | positive regulation of myotube differentiation | Biological process | 2 | 37 | 100 | 17653 | 9.54216216216216 | 0.0186011547423109 | 1 | 1.73046009434945 | 0.02 | NFATC2//CYP26B1// |
| GO:0038095 | Fc-epsilon receptor signaling pathway | Biological process | 4 | 179 | 100 | 17653 | 3.94480446927374 | 0.0188743467949191 | 1 | 1.72412806954095 | 0.04 | NFATC2//MAPK3//MAP2K7//PSMD13// |
| GO:0050868 | negative regulation of T cell activation | Biological process | 3 | 100 | 100 | 17653 | 5.2959 | 0.019195812109819 | 1 | 1.71679350962877 | 0.03 | NFKBID//NDFIP1//CASP3// |
| GO:0042113 | B cell activation | Biological process | 5 | 273 | 100 | 17653 | 3.23315018315018 | 0.0195422719683041 | 1 | 1.70902494696634 | 0.05 | CASP3//NFATC2//ZFP36L1//NDFIP1//IGLL5// |
| GO:0035329 | hippo signaling | Biological process | 2 | 38 | 100 | 17653 | 9.29105263157895 | 0.0195629689394084 | 1 | 1.70856523461202 | 0.02 | TEAD3//CASP3// |
| GO:0002768 | immune response-regulating cell surface receptor signaling pathway | Biological process | 7 | 482 | 100 | 17653 | 2.56371369294606 | 0.0196865066983974 | 1 | 1.70583134105871 | 0.07 | CLEC4C//PSMD13//NFATC2//MAPK3//MAP2K7//WIPF2//IGLL5// |
| GO:0016192 | vesicle-mediated transport | Biological process | 19 | 2050 | 100 | 17653 | 1.63613170731707 | 0.0204904369129091 | 1 | 1.68844878113797 | 0.19 | LRRTM1//F5//SYT16//DYNC1I1//WIPF2//MSR1//PI4KB//CFTR//IGLL5//GRIK5//ARL4C//MAPK3//CLEC4C//ACPP//PSMD13//CLIP3//CAMK1D//SYN1//SMAP1// |
| GO:0009410 | response to xenobiotic stimulus | Biological process | 5 | 277 | 100 | 17653 | 3.18646209386282 | 0.0206631420094783 | 1 | 1.68480363956244 | 0.05 | FMO1//CYP26B1//RORC//CDO1//TFAP4// |
| GO:0017157 | regulation of exocytosis | Biological process | 4 | 186 | 100 | 17653 | 3.79634408602151 | 0.0213881854606083 | 1 | 1.66982605871376 | 0.04 | SYT16//GRIK5//CFTR//SYN1// |
| GO:0009069 | serine family amino acid metabolic process | Biological process | 2 | 40 | 100 | 17653 | 8.8265 | 0.0215478372342093 | 1 | 1.66659631364176 | 0.02 | CDO1//SEPHS2// |
| GO:0033673 | negative regulation of kinase activity | Biological process | 5 | 283 | 100 | 17653 | 3.11890459363958 | 0.0224221980535632 | 1 | 1.64932181565978 | 0.05 | DUSP18//DUSP9//LRRTM1//TFAP4//CASP3// |
| GO:0008219 | cell death | Biological process | 20 | 2216 | 100 | 17653 | 1.59323104693141 | 0.0226233371507469 | 1 | 1.64544333224627 | 0.2 | SHC4//XAF1//TMEM214//MAPK3//MAP2K7//ZFP36L1//CASP3//CAMK1D//GLS2//TNFRSF11B//BMP2//TNFRSF8//CLIP3//TFAP4//GRIK5//NFKBID//CYP26B1//ZMAT4//FAP//SKIL// |
| GO:0030218 | erythrocyte differentiation | Biological process | 3 | 107 | 100 | 17653 | 4.94943925233645 | 0.0229074919180474 | 1 | 1.64002245798561 | 0.03 | ZFP36L1//SMAP1//CASP3// |
| GO:0019218 | regulation of steroid metabolic process | Biological process | 3 | 108 | 100 | 17653 | 4.90361111111111 | 0.0234673672230996 | 1 | 1.62953563066625 | 0.03 | BMP2//ELOVL6//RORC// |
| GO:0060079 | excitatory postsynaptic potential | Biological process | 3 | 108 | 100 | 17653 | 4.90361111111111 | 0.0234673672230996 | 1 | 1.62953563066625 | 0.03 | CHRNA1//GLRB//GRIK5// |
| GO:0048518 | positive regulation of biological process | Biological process | 43 | 5841 | 100 | 17653 | 1.2995702790618 | 0.0240312341788798 | 1 | 1.61922392443899 | 0.43 | DUSP9//MAPK3//MAP2K7//BMP2//CLIP3//CLEC4C//PSMD13//CASP3//IGLL5//ESM1//RTKN2//SHC4//CHN1//CYP26B1//MSR1//CAMK1D//CREBL2//NFATC2//RORC//TFAP4//NFE2L3//NDFIP1//ARFIP1//CFTR//WIPF2//PLS1//TNFRSF8//GRIK5//ZFP36L1//SMAP1//DMRT2//DLX3//TEAD3//BMI1//SKIL//LRRTM1//ACPP//DND1//NFKBID//FAP//GLS2//MIS18A//DPH6// |
| GO:0043200 | response to amino acid | Biological process | 3 | 109 | 100 | 17653 | 4.85862385321101 | 0.0240346524609706 | 1 | 1.61916215335118 | 0.03 | CDO1//GLRB//CASP3// |
| GO:0051153 | regulation of striated muscle cell differentiation | Biological process | 3 | 110 | 100 | 17653 | 4.81445454545455 | 0.0246093460401689 | 1 | 1.60889992692782 | 0.03 | NFATC2//BMP2//CYP26B1// |
| GO:0098693 | regulation of synaptic vesicle cycle | Biological process | 2 | 43 | 100 | 17653 | 8.21069767441861 | 0.0246743998295558 | 1 | 1.60775340213886 | 0.02 | GRIK5//SYN1// |
| GO:0032386 | regulation of intracellular transport | Biological process | 6 | 395 | 100 | 17653 | 2.68146835443038 | 0.0248178178775493 | 1 | 1.60523640677769 | 0.06 | GRIK5//GLS2//MIS18A//DPH6//SYN1//MAPK3// |
| GO:0071900 | regulation of protein serine/threonine kinase activity | Biological process | 7 | 508 | 100 | 17653 | 2.4325 | 0.0253400497253757 | 1 | 1.59619253722546 | 0.07 | DUSP9//MAPK3//MAP2K7//BMP2//DUSP18//TFAP4//CASP3// |
| GO:0070849 | response to epidermal growth factor | Biological process | 2 | 44 | 100 | 17653 | 8.02409090909091 | 0.0257553815252882 | 1 | 1.58913201234911 | 0.02 | ZFP36L1//MAPK3// |
| GO:1903038 | negative regulation of leukocyte cell-cell adhesion | Biological process | 3 | 112 | 100 | 17653 | 4.72848214285714 | 0.0257809480790595 | 1 | 1.58870111576722 | 0.03 | NFKBID//NDFIP1//CASP3// |
| GO:1903749 | positive regulation of establishment of protein localization to mitochondrion | Biological process | 3 | 113 | 100 | 17653 | 4.68663716814159 | 0.026377849612129 | 1 | 1.57876061211233 | 0.03 | GLS2//MIS18A//DPH6// |
| GO:0065008 | regulation of biological quality | Biological process | 30 | 3770 | 100 | 17653 | 1.40474801061008 | 0.0264218926071734 | 1 | 1.57803607697838 | 0.3 | CASP3//SKIL//F5//GRIK5//NDFIP1//SYN1//CDO1//WIPF2//MKS1//MSR1//SYT16//CREBL2//CHRNA1//MAPK3//PLEKHH2//DPH6//BMP2//MAP2K7//PLS1//ARFIP1//CYP26B1//CFTR//KCNH8//GLRB//PSMD13//ZFP36L1//SMAP1//TNFRSF11B//FAP//LRRTM1// |
| GO:0007165 | signal transduction | Biological process | 45 | 6210 | 100 | 17653 | 1.27920289855072 | 0.0265368754742906 | 1 | 1.57615021350464 | 0.45 | KCNH8//DUSP9//MAPK3//PSMD13//ZFP36L1//RASAL2//MAP2K7//BMP2//DUSP18//CLEC4C//SHC4//CHN1//DCX//CAMK1D//TFAP4//VIPR2//REPS2//SKIL//GLRB//CASP3//DDIT4L//TNFRSF11B//CLIP3//LRRTM1//RORC//SMAP1//NFATC2//TNFRSF8//GRIK5//TEAD3//WIPF2//NDFIP1//NFKBID//PI4KB//CYP26B1//IGLL5//CHRNA1//ACPP//XAF1//MKS1//ZMAT4//ESM1//CREBL2//RTKN2//ANKDD1B// |
| GO:0031098 | stress-activated protein kinase signaling cascade | Biological process | 5 | 296 | 100 | 17653 | 2.98192567567568 | 0.0265609215940187 | 1 | 1.57575686016798 | 0.05 | DUSP9//MAP2K7//MAPK3//ZFP36L1//BMP2// |
| GO:0018198 | peptidyl-cysteine modification | Biological process | 2 | 45 | 100 | 17653 | 7.84577777777778 | 0.0268553463963896 | 1 | 1.57096924146125 | 0.02 | CLIP3//ZDHHC11// |
| GO:0045646 | regulation of erythrocyte differentiation | Biological process | 2 | 45 | 100 | 17653 | 7.84577777777778 | 0.0268553463963896 | 1 | 1.57096924146125 | 0.02 | ZFP36L1//SMAP1// |
| GO:0023052 | signaling | Biological process | 48 | 6724 | 100 | 17653 | 1.26017846519929 | 0.0270589646850693 | 1 | 1.56768882415241 | 0.48 | KCNH8//DUSP9//MAPK3//PSMD13//ZFP36L1//RASAL2//MAP2K7//BMP2//DUSP18//CLEC4C//VIPR2//CHRNA1//CREBL2//RTKN2//GLRB//TNFRSF11B//PI4KB//ANKDD1B//NDFIP1//TNFRSF8//SHC4//CHN1//DCX//CAMK1D//TFAP4//REPS2//SKIL//SYN1//CASP3//DDIT4L//CLIP3//SYT16//LRRTM1//RORC//GRIK5//SMAP1//NFATC2//TEAD3//CFTR//WIPF2//NFKBID//CYP26B1//IGLL5//ACPP//XAF1//MKS1//ZMAT4//ESM1// |
| GO:0045786 | negative regulation of cell cycle | Biological process | 8 | 634 | 100 | 17653 | 2.22750788643533 | 0.0274519269286332 | 1 | 1.56142716578215 | 0.08 | SKIL//PSMD13//TFAP4//CASP3//BMI1//FAP//ZFP36L1//BMP2// |
| GO:0034101 | erythrocyte homeostasis | Biological process | 3 | 115 | 100 | 17653 | 4.60513043478261 | 0.0275938309412873 | 1 | 1.5591880008197 | 0.03 | CASP3//ZFP36L1//SMAP1// |
| GO:0038066 | p38MAPK cascade | Biological process | 2 | 46 | 100 | 17653 | 7.67521739130435 | 0.0279740493105923 | 1 | 1.55324466391237 | 0.02 | BMP2//ZFP36L1// |
| GO:0051972 | regulation of telomerase activity | Biological process | 2 | 46 | 100 | 17653 | 7.67521739130435 | 0.0279740493105923 | 1 | 1.55324466391237 | 0.02 | MAPK3//MAP2K7// |
| GO:0050793 | regulation of developmental process | Biological process | 22 | 2563 | 100 | 17653 | 1.51527896995708 | 0.0281567791401178 | 1 | 1.55041702572578 | 0.22 | NDFIP1//BMP2//WIPF2//MSR1//CAMK1D//TNFRSF8//DMRT2//NFKBID//PLS1//TNFRSF11B//ZFP36L1//CYP26B1//NFATC2//PLEKHB2//SKIL//RORC//CREBL2//SMAP1//CHN1//LRRTM1//PSMD13//MKS1// |
| GO:0017156 | calcium ion regulated exocytosis | Biological process | 3 | 116 | 100 | 17653 | 4.56543103448276 | 0.0282128994987596 | 1 | 1.54955227819762 | 0.03 | SYT16//GRIK5//SYN1// |
| GO:0007154 | cell communication | Biological process | 48 | 6744 | 100 | 17653 | 1.25644128113879 | 0.0285759636766614 | 1 | 1.54399911482947 | 0.48 | KCNH8//DUSP9//MAPK3//PSMD13//ZFP36L1//RASAL2//MAP2K7//BMP2//DUSP18//CLEC4C//VIPR2//CHRNA1//CREBL2//RTKN2//GLRB//TNFRSF11B//PI4KB//ANKDD1B//NDFIP1//TNFRSF8//SHC4//CHN1//DCX//CAMK1D//TFAP4//REPS2//SKIL//SYN1//CASP3//DDIT4L//CLIP3//SYT16//LRRTM1//RORC//GRIK5//SMAP1//NFATC2//TEAD3//CFTR//WIPF2//NFKBID//CYP26B1//IGLL5//ACPP//XAF1//MKS1//ZMAT4//ESM1// |
| GO:0035556 | intracellular signal transduction | Biological process | 24 | 2871 | 100 | 17653 | 1.47569487983281 | 0.0289617892136747 | 1 | 1.53817461161774 | 0.24 | KCNH8//DUSP9//MAPK3//PSMD13//ZFP36L1//RASAL2//MAP2K7//BMP2//DUSP18//TFAP4//CASP3//SMAP1//NFATC2//TEAD3//NDFIP1//NFKBID//LRRTM1//PI4KB//CHN1//ZMAT4//SKIL//DCX//SHC4//CAMK1D// |
| GO:0035094 | response to nicotine | Biological process | 2 | 47 | 100 | 17653 | 7.51191489361702 | 0.0291112472545157 | 1 | 1.53593918706721 | 0.02 | CHRNA1//CASP3// |
| GO:0071622 | regulation of granulocyte chemotaxis | Biological process | 2 | 47 | 100 | 17653 | 7.51191489361702 | 0.0291112472545157 | 1 | 1.53593918706721 | 0.02 | MAPK3//CAMK1D// |
| GO:0002694 | regulation of leukocyte activation | Biological process | 7 | 525 | 100 | 17653 | 2.35373333333333 | 0.0295891909983843 | 1 | 1.5288669087745 | 0.07 | CASP3//NFATC2//NFKBID//NDFIP1//ZFP36L1//CYP26B1//IGLL5// |
| GO:0006897 | endocytosis | Biological process | 9 | 766 | 100 | 17653 | 2.07411227154047 | 0.0296213735447107 | 1 | 1.52839480708902 | 0.09 | LRRTM1//MSR1//PI4KB//IGLL5//WIPF2//MAPK3//CLIP3//CAMK1D//SMAP1// |
| GO:0006954 | inflammatory response | Biological process | 9 | 767 | 100 | 17653 | 2.07140808344198 | 0.0298356594489195 | 1 | 1.52526435862894 | 0.09 | RORC//NDFIP1//CDO1//TNFRSF11B//CYP26B1//CAMK1D//BMP2//NFKBID//TNFRSF8// |
| GO:0010828 | positive regulation of glucose transmembrane transport | Biological process | 2 | 48 | 100 | 17653 | 7.35541666666667 | 0.0302666993179229 | 1 | 1.51903493768802 | 0.02 | CREBL2//CLIP3// |
| GO:0043525 | positive regulation of neuron apoptotic process | Biological process | 2 | 48 | 100 | 17653 | 7.35541666666667 | 0.0302666993179229 | 1 | 1.51903493768802 | 0.02 | GRIK5//CASP3// |
| GO:0051640 | organelle localization | Biological process | 8 | 647 | 100 | 17653 | 2.18275115919629 | 0.0304604151927584 | 1 | 1.5162641812792 | 0.08 | CFTR//SYT16//GRIK5//ARL8B//DYNC1I1//F5//SYN1//MKS1// |
| GO:0048489 | synaptic vesicle transport | Biological process | 3 | 120 | 100 | 17653 | 4.41325 | 0.0307628630580107 | 1 | 1.51197324779023 | 0.03 | SYT16//GRIK5//SYN1// |
| GO:0097480 | establishment of synaptic vesicle localization | Biological process | 3 | 120 | 100 | 17653 | 4.41325 | 0.0307628630580107 | 1 | 1.51197324779023 | 0.03 | SYT16//GRIK5//SYN1// |
| GO:0051348 | negative regulation of transferase activity | Biological process | 5 | 309 | 100 | 17653 | 2.85647249190939 | 0.0311602073601257 | 1 | 1.50639966091415 | 0.05 | DUSP18//DUSP9//LRRTM1//TFAP4//CASP3// |
| GO:0034097 | response to cytokine | Biological process | 12 | 1164 | 100 | 17653 | 1.81989690721649 | 0.0315263722122602 | 1 | 1.50132600130546 | 0.12 | CLIP3//LRRTM1//RORC//CASP3//TNFRSF11B//PSMD13//TNFRSF8//MAP2K7//XAF1//MAPK3//ZFP36L1//SKIL// |
| GO:0008202 | steroid metabolic process | Biological process | 5 | 311 | 100 | 17653 | 2.83810289389068 | 0.0319095246978957 | 1 | 1.4960796647071 | 0.05 | CFTR//BMP2//CYP26B1//RORC//ELOVL6// |
| GO:1903305 | regulation of regulated secretory pathway | Biological process | 3 | 122 | 100 | 17653 | 4.34090163934426 | 0.0320819394098123 | 1 | 1.49373938572701 | 0.03 | SYT16//GRIK5//SYN1// |
| GO:0043113 | receptor clustering | Biological process | 2 | 50 | 100 | 17653 | 7.0612 | 0.0326314125842497 | 1 | 1.48636412559103 | 0.02 | GLRB//GRIK5// |
| GO:0042476 | odontogenesis | Biological process | 3 | 123 | 100 | 17653 | 4.30560975609756 | 0.0327524669392855 | 1 | 1.48475598306855 | 0.03 | DLX3//BMP2//TNFRSF11B// |
| GO:0099565 | chemical synaptic transmission, postsynaptic | Biological process | 3 | 124 | 100 | 17653 | 4.27088709677419 | 0.0334303059636832 | 1 | 1.47585964860243 | 0.03 | CHRNA1//GLRB//GRIK5// |
| GO:0051641 | cellular localization | Biological process | 23 | 2761 | 100 | 17653 | 1.47055052517204 | 0.033755735121037 | 1 | 1.47165242965313 | 0.23 | ZDHHC11//GRIK5//ARFIP1//DYNC1I1//F5//CFTR//SYN1//SYT16//ARL8B//ARL4C//TRAM2//WIPF2//PRPF18//GLRB//MKS1//CLIP3//PLS1//GLS2//MIS18A//DPH6//RTKN2//ZFP36L1//MAPK3// |
| GO:0032206 | positive regulation of telomere maintenance | Biological process | 2 | 51 | 100 | 17653 | 6.92274509803922 | 0.0338402023422237 | 1 | 1.47056704882898 | 0.02 | MAPK3//MAP2K7// |
| GO:0045010 | actin nucleation | Biological process | 2 | 51 | 100 | 17653 | 6.92274509803922 | 0.0338402023422237 | 1 | 1.47056704882898 | 0.02 | ARFIP1//WIPF2// |
| GO:0071320 | cellular response to cAMP | Biological process | 2 | 51 | 100 | 17653 | 6.92274509803922 | 0.0338402023422237 | 1 | 1.47056704882898 | 0.02 | CFTR//ZFP36L1// |
| GO:1903747 | regulation of establishment of protein localization to mitochondrion | Biological process | 3 | 126 | 100 | 17653 | 4.20309523809524 | 0.0348078706186592 | 1 | 1.45832254399099 | 0.03 | GLS2//MIS18A//DPH6// |
| GO:1903078 | positive regulation of protein localization to plasma membrane | Biological process | 2 | 52 | 100 | 17653 | 6.78961538461539 | 0.0350663032990495 | 1 | 1.45511001516911 | 0.02 | CLIP3//PLS1// |
| GO:0097479 | synaptic vesicle localization | Biological process | 3 | 127 | 100 | 17653 | 4.17 | 0.0355075708207233 | 1 | 1.44967903806847 | 0.03 | SYT16//GRIK5//SYN1// |
| GO:1903533 | regulation of protein targeting | Biological process | 3 | 127 | 100 | 17653 | 4.17 | 0.0355075708207233 | 1 | 1.44967903806847 | 0.03 | GLS2//MIS18A//DPH6// |
| GO:0050863 | regulation of T cell activation | Biological process | 5 | 321 | 100 | 17653 | 2.74968847352025 | 0.0358256501949757 | 1 | 1.44580591890612 | 0.05 | NFKBID//NDFIP1//CYP26B1//NFATC2//CASP3// |
| GO:0045580 | regulation of T cell differentiation | Biological process | 3 | 128 | 100 | 17653 | 4.137421875 | 0.0362145316799566 | 1 | 1.44111712664788 | 0.03 | NFKBID//NFATC2//CYP26B1// |
| GO:0032210 | regulation of telomere maintenance via telomerase | Biological process | 2 | 53 | 100 | 17653 | 6.66150943396226 | 0.0363094848277959 | 1 | 1.43997991297179 | 0.02 | MAPK3//MAP2K7// |
| GO:0009743 | response to carbohydrate | Biological process | 4 | 220 | 100 | 17653 | 3.20963636363636 | 0.0364432687563755 | 1 | 1.43838267617792 | 0.04 | CASP3//CFTR//GRIK5//ZFP36L1// |
| GO:1901605 | alpha-amino acid metabolic process | Biological process | 4 | 220 | 100 | 17653 | 3.20963636363636 | 0.0364432687563755 | 1 | 1.43838267617792 | 0.04 | CDO1//GLS2//SEPHS2//AGMAT// |
| GO:0060078 | regulation of postsynaptic membrane potential | Biological process | 3 | 129 | 100 | 17653 | 4.1053488372093 | 0.036928739036071 | 1 | 1.43263552145884 | 0.03 | CHRNA1//GLRB//GRIK5// |
| GO:0019221 | cytokine-mediated signaling pathway | Biological process | 9 | 798 | 100 | 17653 | 1.99093984962406 | 0.0370320966148729 | 1 | 1.43142169917136 | 0.09 | CLIP3//TNFRSF11B//PSMD13//TNFRSF8//XAF1//MAPK3//LRRTM1//RORC//CASP3// |
| GO:0007049 | cell cycle | Biological process | 17 | 1898 | 100 | 17653 | 1.58114330874605 | 0.0375118317746479 | 1 | 1.42583172789946 | 0.17 | RTKN2//YY1AP1//SKIL//DYNC1I1//PSMD13//CYP26B1//MAP2K7//TFAP4//CASP3//BMP2//BMI1//FAP//ZFP36L1//CREBL2//MIS18A//ARL8B//MAPK3// |
| GO:0032496 | response to lipopolysaccharide | Biological process | 5 | 327 | 100 | 17653 | 2.69923547400612 | 0.0383122355676313 | 1 | 1.41666250564263 | 0.05 | MAPK3//FMO1//TNFRSF11B//CASP3//TNFRSF8// |
| GO:0046683 | response to organophosphorus | Biological process | 3 | 131 | 100 | 17653 | 4.04267175572519 | 0.038378833799969 | 1 | 1.4159082261074 | 0.03 | CDO1//CFTR//ZFP36L1// |
| GO:0045595 | regulation of cell differentiation | Biological process | 16 | 1761 | 100 | 17653 | 1.60390687109597 | 0.0387017898762199 | 1 | 1.41226894931209 | 0.16 | NDFIP1//BMP2//MSR1//CAMK1D//TNFRSF8//NFKBID//ZFP36L1//CYP26B1//NFATC2//SKIL//RORC//CREBL2//SMAP1//CHN1//PSMD13//PLEKHB2// |
| GO:0016233 | telomere capping | Biological process | 2 | 55 | 100 | 17653 | 6.41927272727273 | 0.0388461771329063 | 1 | 1.41065171384567 | 0.02 | MAPK3//MAP2K7// |
| GO:0042130 | negative regulation of T cell proliferation | Biological process | 2 | 55 | 100 | 17653 | 6.41927272727273 | 0.0388461771329063 | 1 | 1.41065171384567 | 0.02 | CASP3//NDFIP1// |
| GO:0046928 | regulation of neurotransmitter secretion | Biological process | 2 | 55 | 100 | 17653 | 6.41927272727273 | 0.0388461771329063 | 1 | 1.41065171384567 | 0.02 | GRIK5//SYN1// |
| GO:0060563 | neuroepithelial cell differentiation | Biological process | 2 | 55 | 100 | 17653 | 6.41927272727273 | 0.0388461771329063 | 1 | 1.41065171384567 | 0.02 | BMP2//DLX3// |
| GO:0051250 | negative regulation of lymphocyte activation | Biological process | 3 | 132 | 100 | 17653 | 4.01204545454545 | 0.0391146901171093 | 1 | 1.40766010606637 | 0.03 | CASP3//NFKBID//NDFIP1// |
| GO:0001101 | response to acid chemical | Biological process | 5 | 329 | 100 | 17653 | 2.68282674772037 | 0.0391641053647579 | 1 | 1.40711178970319 | 0.05 | SLC22A6//CDO1//GLRB//CASP3//CYP26B1// |
| GO:0006909 | phagocytosis | Biological process | 5 | 330 | 100 | 17653 | 2.67469696969697 | 0.0395943699513752 | 1 | 1.40236656338965 | 0.05 | IGLL5//MSR1//WIPF2//MAPK3//CAMK1D// |
| GO:0071322 | cellular response to carbohydrate stimulus | Biological process | 3 | 133 | 100 | 17653 | 3.98187969924812 | 0.0398577307681174 | 1 | 1.39948743074688 | 0.03 | CFTR//GRIK5//ZFP36L1// |
| GO:0099504 | synaptic vesicle cycle | Biological process | 3 | 133 | 100 | 17653 | 3.98187969924812 | 0.0398577307681174 | 1 | 1.39948743074688 | 0.03 | SYT16//GRIK5//SYN1// |
| GO:0050865 | regulation of cell activation | Biological process | 7 | 563 | 100 | 17653 | 2.19486678507993 | 0.0407768146794327 | 1 | 1.38958670256905 | 0.07 | CASP3//NFATC2//NFKBID//NDFIP1//ZFP36L1//CYP26B1//IGLL5// |
| GO:0046649 | lymphocyte activation | Biological process | 8 | 686 | 100 | 17653 | 2.05865889212828 | 0.0408368720220579 | 1 | 1.38894753092366 | 0.08 | CYP26B1//CASP3//NFATC2//ZFP36L1//NFKBID//NDFIP1//IGLL5//RORC// |
| GO:0003002 | regionalization | Biological process | 5 | 333 | 100 | 17653 | 2.6506006006006 | 0.0409025207732573 | 1 | 1.38824992612027 | 0.05 | BMI1//CYP26B1//DMRT2//BMP2//MKS1// |
| GO:0030100 | regulation of endocytosis | Biological process | 4 | 229 | 100 | 17653 | 3.08349344978166 | 0.0412374431431419 | 1 | 1.38470827023743 | 0.04 | LRRTM1//CLIP3//CAMK1D//SMAP1// |
| GO:0002429 | immune response-activating cell surface receptor signaling pathway | Biological process | 6 | 450 | 100 | 17653 | 2.35373333333333 | 0.042696564255388 | 1 | 1.36960707075857 | 0.06 | CLEC4C//PSMD13//WIPF2//MAPK3//IGLL5//NFATC2// |
| GO:1904377 | positive regulation of protein localization to cell periphery | Biological process | 2 | 58 | 100 | 17653 | 6.08724137931034 | 0.0427736690345338 | 1 | 1.36882349528658 | 0.02 | CLIP3//PLS1// |
| GO:0051130 | positive regulation of cellular component organization | Biological process | 12 | 1221 | 100 | 17653 | 1.73493857493857 | 0.0430232340242922 | 1 | 1.36629694713045 | 0.12 | CAMK1D//MAPK3//MAP2K7//ARFIP1//CLIP3//SKIL//WIPF2//LRRTM1//NFATC2//GLS2//MIS18A//DPH6// |
| GO:0071407 | cellular response to organic cyclic compound | Biological process | 7 | 572 | 100 | 17653 | 2.16033216783217 | 0.0437832773650638 | 1 | 1.35869173275691 | 0.07 | RORC//CFTR//ZFP36L1//TFAP4//CASP3//MAPK3//BMP2// |
| GO:0002262 | myeloid cell homeostasis | Biological process | 3 | 139 | 100 | 17653 | 3.81 | 0.0444658650272251 | 1 | 1.35197325461569 | 0.03 | CASP3//ZFP36L1//SMAP1// |
| GO:0099003 | vesicle-mediated transport in synapse | Biological process | 3 | 139 | 100 | 17653 | 3.81 | 0.0444658650272251 | 1 | 1.35197325461569 | 0.03 | SYT16//GRIK5//SYN1// |
| GO:0071242 | cellular response to ammonium ion | Biological process | 2 | 60 | 100 | 17653 | 5.88433333333333 | 0.045471057413409 | 1 | 1.3422649463124 | 0.02 | CASP3//MAPK3// |
| GO:1904356 | regulation of telomere maintenance via telomere lengthening | Biological process | 2 | 60 | 100 | 17653 | 5.88433333333333 | 0.045471057413409 | 1 | 1.3422649463124 | 0.02 | MAPK3//MAP2K7// |
| GO:0043405 | regulation of MAP kinase activity | Biological process | 5 | 344 | 100 | 17653 | 2.56584302325581 | 0.0459228922622001 | 1 | 1.33797076751866 | 0.05 | DUSP9//MAPK3//MAP2K7//BMP2//DUSP18// |
| GO:0048872 | homeostasis of number of cells | Biological process | 4 | 238 | 100 | 17653 | 2.96689075630252 | 0.046373904721618 | 1 | 1.33372633460546 | 0.04 | CASP3//SKIL//ZFP36L1//SMAP1// |
| GO:0002237 | response to molecule of bacterial origin | Biological process | 5 | 345 | 100 | 17653 | 2.55840579710145 | 0.0463968017434465 | 1 | 1.33351195550212 | 0.05 | MAPK3//FMO1//TNFRSF11B//CASP3//TNFRSF8// |
| GO:0001824 | blastocyst development | Biological process | 2 | 61 | 100 | 17653 | 5.78786885245902 | 0.0468428191327582 | 1 | 1.32935697574831 | 0.02 | SKIL//GINS4// |
| GO:0010830 | regulation of myotube differentiation | Biological process | 2 | 61 | 100 | 17653 | 5.78786885245902 | 0.0468428191327582 | 1 | 1.32935697574831 | 0.02 | NFATC2//CYP26B1// |
| GO:0030888 | regulation of B cell proliferation | Biological process | 2 | 61 | 100 | 17653 | 5.78786885245902 | 0.0468428191327582 | 1 | 1.32935697574831 | 0.02 | CASP3//NFATC2// |
| GO:0071385 | cellular response to glucocorticoid stimulus | Biological process | 2 | 61 | 100 | 17653 | 5.78786885245902 | 0.0468428191327582 | 1 | 1.32935697574831 | 0.02 | TFAP4//ZFP36L1// |
| GO:1903829 | positive regulation of cellular protein localization | Biological process | 5 | 347 | 100 | 17653 | 2.54365994236311 | 0.047353404428386 | 1 | 1.32464879234577 | 0.05 | CLIP3//PLS1//GLS2//MIS18A//DPH6// |
| GO:0051128 | regulation of cellular component organization | Biological process | 20 | 2403 | 100 | 17653 | 1.46924677486475 | 0.0478298247984078 | 1 | 1.32030120995438 | 0.2 | ESM1//LRRTM1//WIPF2//FAP//CAMK1D//TNFRSF8//PLEKHH2//CLIP3//GRIK5//MAPK3//MAP2K7//PLS1//ARFIP1//CHN1//SKIL//NFATC2//GLS2//MIS18A//DPH6//SMAP1// |
| GO:0048645 | animal organ formation | Biological process | 2 | 62 | 100 | 17653 | 5.69451612903226 | 0.0482296744937329 | 1 | 1.31668566915945 | 0.02 | MAPK3// |
| GO:0016043 | cellular component organization | Biological process | 44 | 6269 | 100 | 17653 | 1.23900462593715 | 0.0485540371210807 | 1 | 1.31377465398429 | 0.44 | WIPF2//PRPF18//RTKN2//ESM1//LRRTM1//PLS1//ABLIM2//SYT16//CASP3//IGLL5//MSR1//DYNC1I1//PSMD13//CYP26B1//MAPK3//TNFRSF8//CHRNA1//CHN1//MKS1//FAP//CAMK1D//TNFRSF11B//PLEKHH2//CLIP3//GRIK5//MAP2K7//GINS4//MIS18A//ARFIP1//BMI1//NIP7//SLC22A6//TRAM2//F5//SKIL//ACPP//GLRB//CFTR//REPS2//TFAP4//NFATC2//GLS2//DPH6//SMAP1// |
| GO:0007269 | neurotransmitter secretion | Biological process | 3 | 145 | 100 | 17653 | 3.65234482758621 | 0.0493279547702309 | 1 | 1.30690689083051 | 0.03 | SYT16//GRIK5//SYN1// |
| GO:0099643 | signal release from synapse | Biological process | 3 | 145 | 100 | 17653 | 3.65234482758621 | 0.0493279547702309 | 1 | 1.30690689083051 | 0.03 | SYN1//SYT16//GRIK5// |
| GO:0002260 | lymphocyte homeostasis | Biological process | 2 | 63 | 100 | 17653 | 5.60412698412698 | 0.0496314123155427 | 1 | 1.30424336630741 | 0.02 | CASP3//SKIL// |
| GO:1901607 | alpha-amino acid biosynthetic process | Biological process | 2 | 63 | 100 | 17653 | 5.60412698412698 | 0.0496314123155427 | 1 | 1.30424336630741 | 0.02 | GLS2//SEPHS2// |
